# Supplementary material for: Efficacy of L-Arabinose in Lowering Glycemic and Insulinemic Responses: The Modifying Effect of Starch and Fat
Source: Foods. 2022 Jan 8;11(2):157. doi: 10.3390/foods11020157 (PMC8774789; doi:10.3390/foods11020157)
Supplement: Supplementary file 1 [file foods-11-00157-s001.zip › foods-1494256-supplementary.pdf]

Supplemental data - Efficacy of L-arabinose in lowering glycemic and insulinemic responses: the modifying effect of starch and fat Pol, Puhlmann, Mars, Foods 2022.

Supplemental data S1 results *in vitro* experimenting for the preparation of the test drinks

In vitro digestion assessment of model drinks

Before the human interventions study a fat and a starch model drink was designed and tested in an *in vitro* digestion experiment for stability under acid gastric conditions and possible viscosity changes during oral and gastric digestion, respectively. An adapted version of the INFOGEST model was used for *in vitro* digestion assessment. The small intestinal phase was omitted, as we were only interested in oral and gastric changes, and dynamic conditions for gastric acidification were included to reliable mimic remaining amylase activity at higher gastric pH values and to expose the oil droplets to gradual pH changes similar to the *in vivo* situation. Moreover, the amount of simulated salivary fluid (SSF) was based on dry matter, which is also closer to the saliva secretion in the *in vivo* situation when consuming liquids. Finally, a lipase was added during the gastric digestion to create a physiologically more relevant environment gastric stability testing of the fat model drink.

INFOGEST protocol

The preparation of the simulated digestive salivary (SSF) and gastric fluids (SGF) is given in Table S1.1 including the adaptions for gradual gastric acidification.

Table S1.1 Composition of simulated digestive fluids based on the INFOGEST protocol for simulated salivary fluid (SSF) and simulated gastric fluid (SGF). SSF and SGF were prepared as two times concentrated stocks without calcium chloride. Calcium chloride stock was added only prior to the experiment into the final SSF and SGF as advised by Infogest. The pH of SGF was changed to neutral due to the implication of a dynamic addition of SGF during the gastric phase.

Electrolyte Stocks			SSF stock at pH 7		SGF stock pH7	
			Volume electrolyte stock	Concentration in SSF stock	Volume electrolyte stock	Concentration in SGF stock
	g/l	mol/l	ml	mmol/l	ml	mmol/l
**KCl**	37.3	0.5	30.2	30.2	6.9	13.8
**KH_2PO_4**	68	0.5	7.4	7.4	0.9	1.8
**$NaHCO_3$**	84	1	13.6	27.2	12.5	50
**NaCl**	117	2			11.8	94.4
**$MgCl_2(H_2O)_6$**	30.5	0.15	1	0.3	0.4	0.24
**$(NH_4)_2CO_3$**	48	0.5	0.12	0.12	0.5	1
**$CaCl_2(H_20)_2$**	44.1	0.3		3		0.3

The different electrolyte solutions were prepared separately and then combined and adjusted to pH 7 to create a 2x concentrated SSF and SGF stock. Prior to each experiment, SSF and SGF were diluted and the pH of SGF was reduced to pH 2. Alpha-amylase from Bacillus lichenformis (Sigma A3403) was used to mimic the action of salivary amylase during the oral phase. This alpha-amylase has a pH optimum slightly lower than human salivary amylase with pH 6-6.5 vs pH 7, but expected to sufficiently mimic

starch degradation in this model. It was supplied in a liquid form with 20100U/ml, which was used as amylase stock. Lipase from Rhizopus oryzae (Sigma 80612) was used to mimic lipolytic action during the gastric digestion phase. Prior to the experiment lipase was dissolved in cold millipore water to a stock solution of 352 FIP/ml. Both enzymes stocks were kept on ice until usage. Experiments were performed in a jacketed beaker kept at 37°C and placed on a magnetic stirring plate set at medium SPEED 3 (IKA, Netherlands). Each beaker was connected to a lab peristaltic pump (SimDos 02®, KNF, Netherlands), which was prefilled with SGF. The *in vitro* digestion consisted of 2 min oral phase at 37°C (final amylase concentration 75 U/mL) followed by 120 min gastric phase at 37°C with a pH gradient decreasing to pH 2 (final lipase concentration 4.3 FIP/ml). A tenth of the SGF was added at the beginning of the gastric phase as baseline secretion to mimic the gastric juices already present in the stomach. The peristaltic pumps were pre-programmed to a secretion rate based on the remaining amount of SGF to be added divided by the time of the gastric phase (120min). The SGF added at the beginning of the gastric phase, and the samples were heated to 37°C prior to the experiment. *In vitro* digestion were performed in triplicate, and samples were analysed at t=0 (prior to the start of the oral phase) and at t = 2, 32, 62 and 122min. For the starch model drink the pH was lowered to pH 2 using 6M HCl at the end of the oral phase to inhibit remaining amylase activity that would have interfered with viscosity measurements.

Stability of fat model drink during *in vitro* digestion assessment

Two fat model drinks were produced using Tween80 to create oil-in-water emulsions, with the aim to be stable under acid gastric conditions. Gastric stability was confirmed during an *in vitro* digestion using a dynamic gastric pH decrease from 6.4 (beginning of gastric phase, measured after addition of SGF baseline secretion) to 2.0 within 120min To follow the stability of the fat model drink (the oil-in-water emulsion) droplet sizes measurements were done using laser light scattering (Mastersizer, Malver 3000, Malvern Instruments, United Kingdom) with the refractive index of the dispersed phase (sunflower oil) set to 1.46 and for the continuous phase (water) set to 1.33. Moreover, an inverted light microscope (Axio Scope A.1, Zeiss, Germany) was used to visually observe changes in emulsion stability. No changes in droplet size distribution (Figure S1.1) and no visual changes (Figure S1.2) were observed, which indicates that the oil droplets remained stable under acid gastric conditions without phase separation.

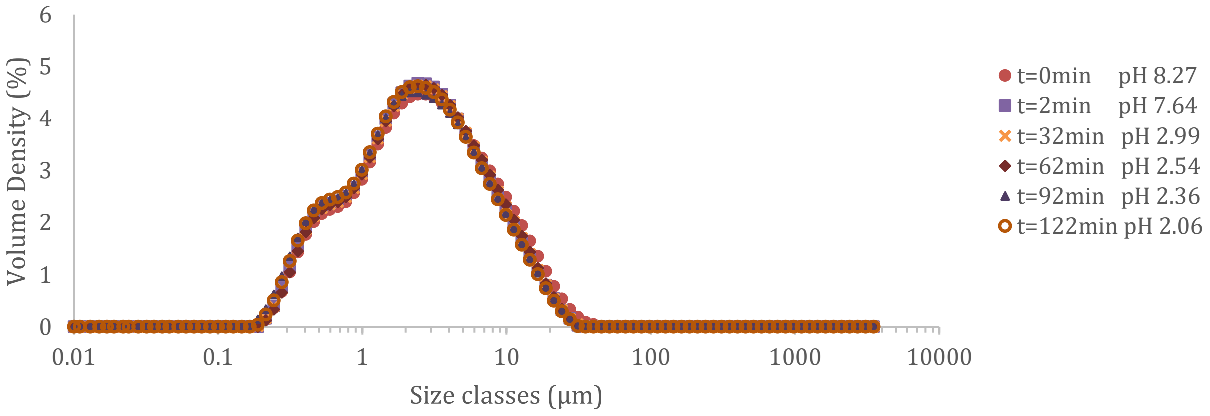

Figure S1.1 Droplet size distributions of fat treatments before and during oral (2min) and gastric (30, 60, 90 and 120min) *in vitro* digestion. Each digestion was performed in triplicate and measurements per time point were repeated five times. Results represent the average of all measurements per time point.

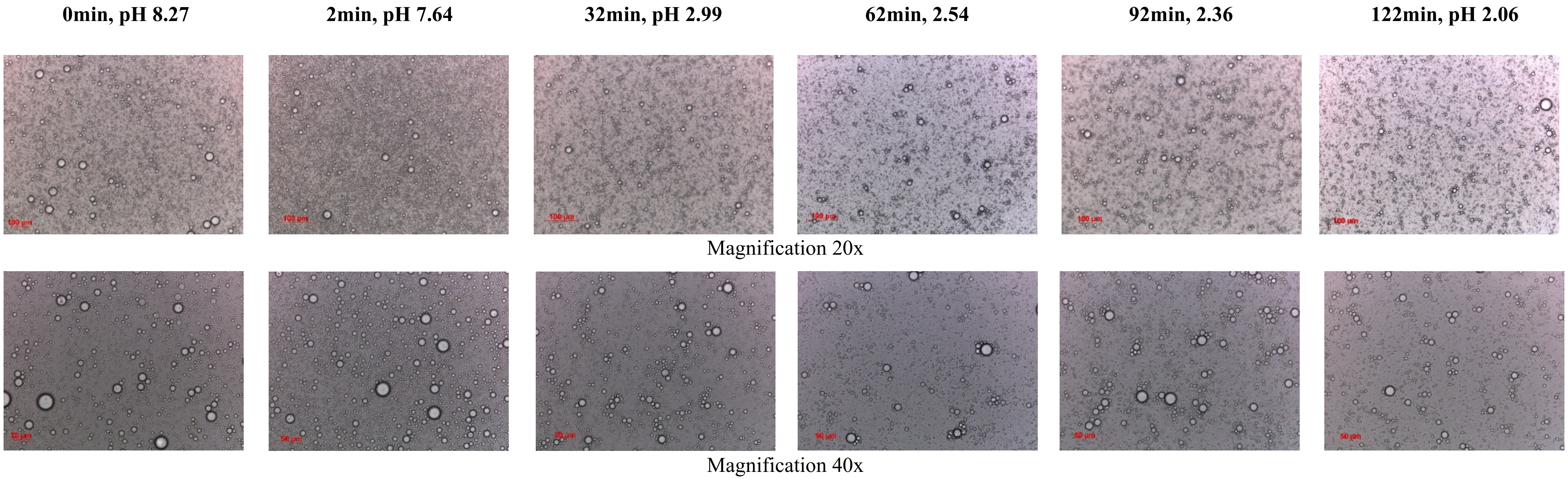

Figure S1.2 Microscopic pictures of fat model drinks before and during oral (t = 2min) and gastric (t = 30, 60, 90 and 120min) *in vitro* digestion.

Decrease in viscosity of starch model drink *in vitro* digestion assessment

A model starch drink was developed using pre-gelatinized starch to dissolve starch easily and avoid possible changes in concentration during heating. As starch increases the viscosity of foods and viscosity is known to impact further digestion (e.g. transit time), changes in viscosity of the starch drink were followed using a rheometers (Anton Paar, MCR 301 and 502, Netherlands). A cone-plate geometry was used and samples were measured in duplicate at 37°C while logarithmically increasing the shear rate from $\dot{\gamma}$ = 0.1 to 100 s^{-1} and then decreasing from $\dot{\gamma}$ = 100 to 0.1 s^{-1}. Due to sensibility issues below shear rates of 10 s^{-1} only data with reliable torque values for the range of 10 – 100 s^{-1} were used for analysis.

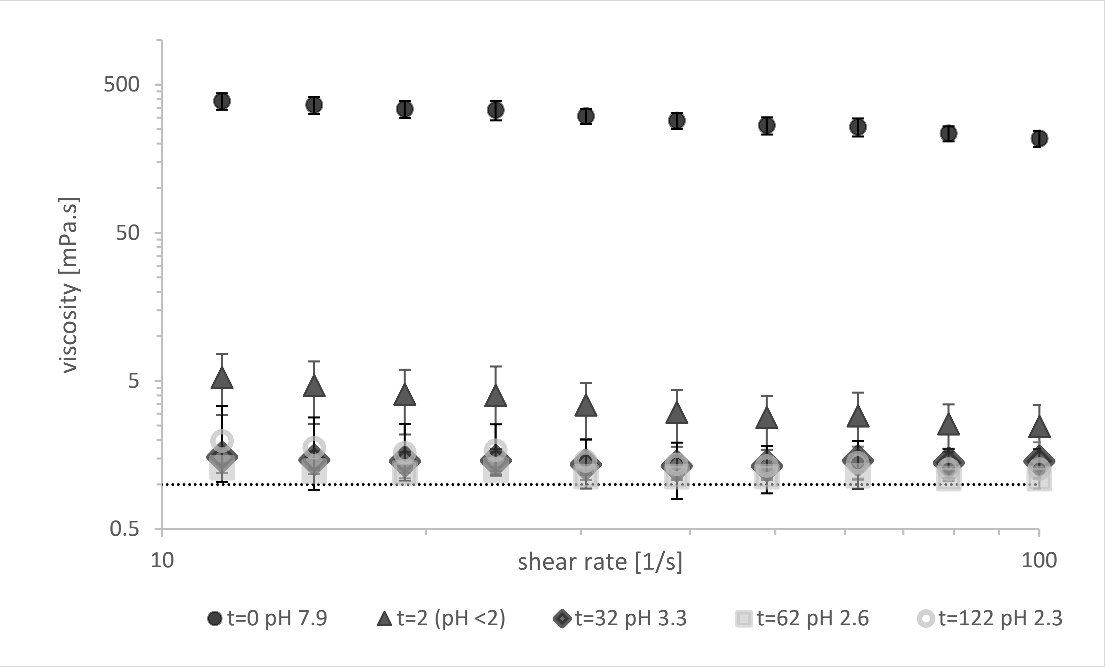

Figure S1.3 Rheology profiles at 37°C of starch solutions before (t=0) and during oral (t=2) and gastric (t = 30, 60 and 120min) *in vitro* digestion with corresponding pH values. The pH at end of the oral phase (t=2) was lowered from 7.9 to below pH 2 to inhibit remaining amylase. Results represent the averages of the viscosity measurements performed on all three repetitions of the *in vitro* digestion experiments for the treatments. The fat treatment had on a viscosity of 0.8 mPa.s at all shear rates (data not shown). For reference, the and the viscosity of water is indicated at 1 mPa.s.

The undigested starch model drink had a viscosity of 200 – 400 mPa.s. We observed large variability in viscosity measurements for the end of the oral phase, which was likely due to remaining amylase activity at pH 7.9 and resulting changes in starch molecule lengths during the measurement. Therefore, we inhibited the remaining amylase activity of the samples prior to the measurement by decreasing the pH to below pH 2 to achieve reliable measurements. Already within the two minute of oral *in vitro* digestion with amylase the viscosity rapidly dropped to 2 - 4 mPa.s and further decreased to 1 – 2 mPa.s during the gastric *in vitro* digestion, which is close to the viscosity of water (about 1mPa.s) and that of the fat treatment with 0.8 mPa.s (at all shear rates). This indicates that the starch and fat model drinks were likely to be of similar viscosity when entering the small intestine and hence viscosity was unlikely to impact further digestion.

Supplemental Data S2 Subjective appetite parameters, thirst and comfort.

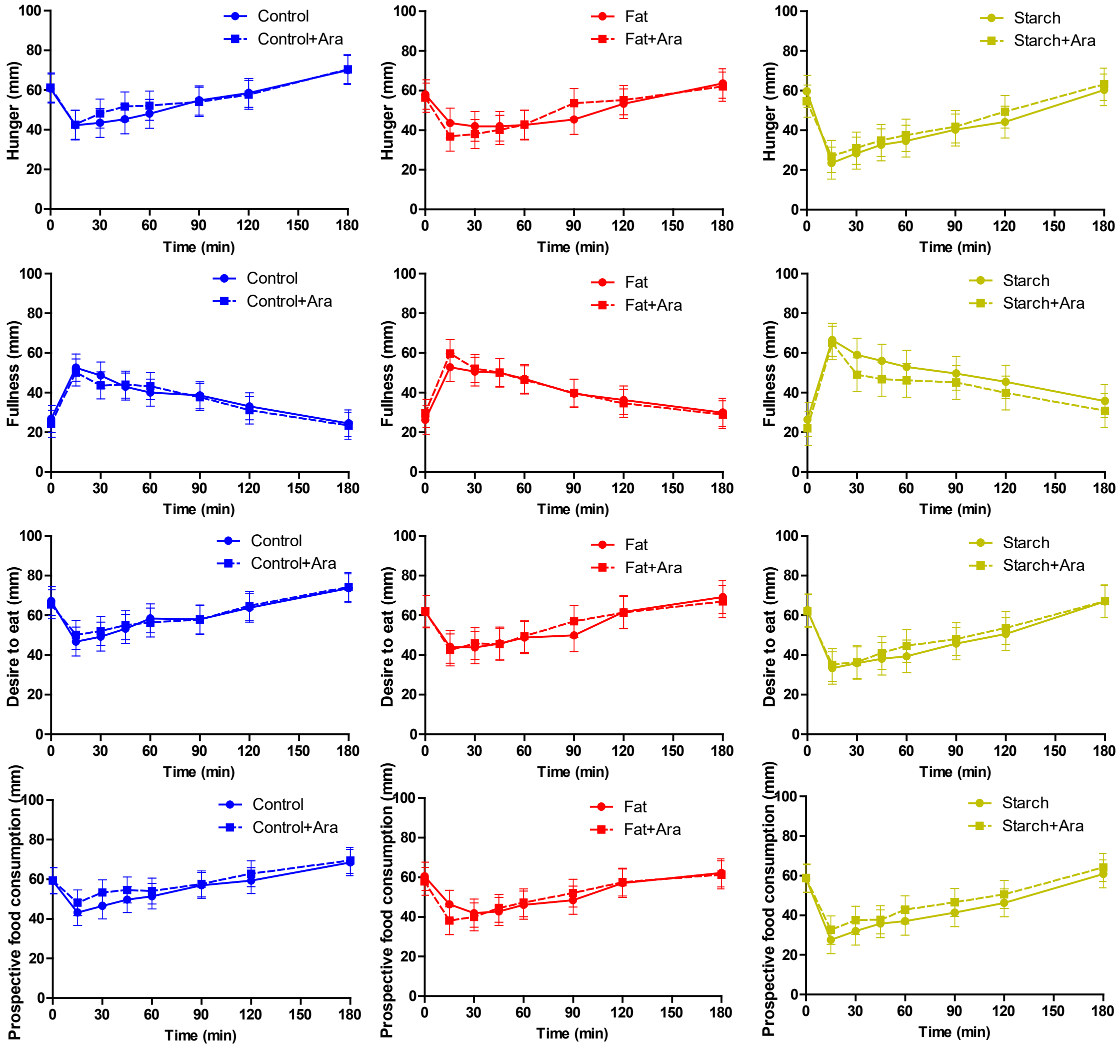

Figure S2.1 Least square means (± 95% confidence interval) subjective hunger, fullness, desire to eat, and prospective food consumption, measured up to 180 minutes postprandial on a 100 mm VAS scale, after consumption of the experimental drinks containing either: 50g sucrose (Control); 50g sucrose and 5g L-arabinose (Control+Ara); 50g sucrose and 22g oil (Fat); 50g sucrose, 5g L-arabinose and 22g oil (Fat+Ara); 50g sucrose and 50g starch (Starch); 50g sucrose, 5g L-arabinose and 50g starch (Starch+Ara) in healthy subjects (n=23). None of the comparisons were significant different for the time×treatment interaction ($P>0.05$); all time effects were significant (all $P<0.001$); the treatment effect was significant for prospective food consumption for the control drinks ($P=0.03$), and for fullness and prospective food consumption for the starch drinks ($P=0.001$ and $P=0.01$ respectively).

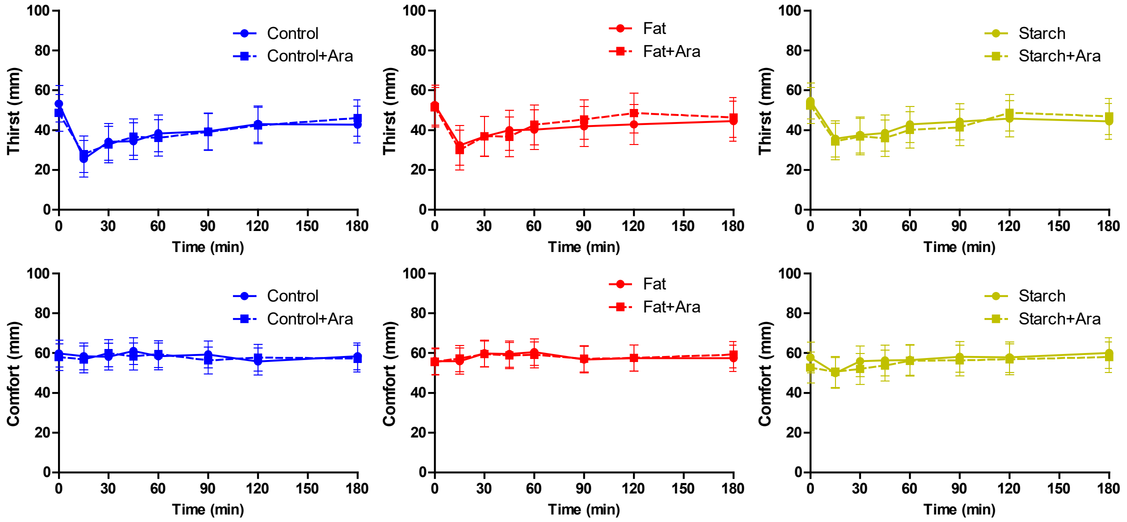

Figure S2.2 Least square means (± 95% confidence interval) subjective thirst and comfort feelings measured up to 180 minutes postprandial on a 100 mm VAS scale, after consumption of the experimental drinks containing either: 50g sucrose (Control); 50g sucrose and 5g L-arabinose (Control+Ara); 50g sucrose and 22g oil (Fat); 50g sucrose, 5g L-arabinose and 22g oil (Fat+Ara); 50g sucrose and 50g starch (Starch); 50g sucrose, 5g L-arabinose and 50g starch (Starch+Ara) in healthy subjects (n=23). None of the comparisons were significant different for the time×treatment interaction ($P>0.05$), nor for the treatment effect ($P>0.05$); time effects were significant for thirst ($P<0.001$), not for comfort feelings.

Supplemental Data S3 Food intake

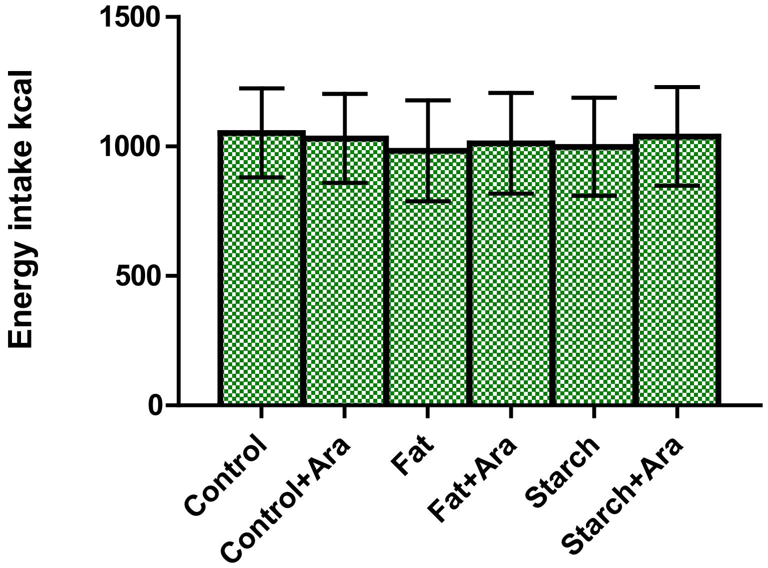

Figure S3 Least square means (± 95% confidence interval) of energy intake after subsequent ad libitum lunch. All P>0.05.

Supplemental Data S4 Gastro-intestinal comfort

Table S4. Change in gastrointestinal symptoms from baseline to 180 minutes postprandial after a single dose of sucrose water with or without L-arabinose and with or without fat or starch (LSMEANS (95% CI)). Measured on a 4-point scale, where 0=None, 1=Little, 2=Modest, 3=Severe.

	**Standard**	**Standard + L-Arabinose**	**P-value***
**Control**			
Δ bloating	-0.17 (-0.42 – 0.08)	-0.17 (-0.42 – 0.08)	1.00
Δ regurgitation	0.00 (-0.09 – 0.09)	0.00 (-0.09 – 0.09)	1.00
Δ nausea	-0.09 (-0.28 – 0.11)	0.17 (-0.02 – 0.37)	0.06
Δ flatulence	-0.17 (-0.32 - -0.02)	0.00 (-0.15 – 0.15)	0.10
**Fat**			
Δ bloating	0.28 (-0.02 – 0.58)	0.30 (0.01 – 0.60)	0.83
Δ regurgitation	0.04 (-0.17 – 0.26)	0.17 (-0.04 – 0.39)	0.28
Δ nausea	0.18 (-0.14 – 0.50)	0.04 (-0.67 – 0.36)	0.56
Δ flatulence	-0.09 (-0.35 – 0.17)	-0.13 (-0.38 – 0.12)	0.81
**Starch**			
Δ bloating	0.09 (-0.10 – 0.27)	0.14 (-0.05 – 0.33)	0.27
Δ regurgitation	0.17 (-0.06 – 0.41)	0.18 (-0.06 – 0.42)	0.96
Δ nausea	0.13 (-0.09 – 0.35)	0.14 (-0.09 – 0.36)	0.97
Δ flatulence	-0.04 (-0.26 – 0.17)	0.00 (-0.22 – 0.22)	0.67
